# Massive Osteolysis of the Mandible in Gorham’s Disease: Our Case Experience

**DOI:** 10.7759/cureus.81063

**Published:** 2025-03-24

**Authors:** Balaji Jayaraman, Arunkumar Kamalakaran, Dakshayani Balaji, Kamalakannan Padmanaban, Vignesh Senthilkumar, Naif Ahamed

**Affiliations:** 1 Oral and Maxillofacial Surgery, Tamil Nadu Government Dental College and Hospital, Chennai, IND; 2 Public Health Dentistry, Sri Ramachandra Dental College and Hospital, Sri Ramachandra Institute of Higher Education and Research, Chennai, IND

**Keywords:** disappearing bone disease, gorham’s disease, idiopathic osteolysis, massive osteolysis, phantom bone disease, spontaneous bone resorption, vanishing bone disease

## Abstract

Vanishing bone disease is an exceptionally rare condition marked by the progressive loss of bone, ultimately leading to its complete disappearance. The cause of this disease remains unknown, and it primarily affects the axial skeleton, pelvis, and humerus. Due to its atypical presentation and rarity, it is often mistaken for other disorders, making an accurate diagnosis challenging and typically a process of elimination. Gorham’s disease has been documented in the maxillofacial region, with most involving the mandible. We present a case involving a 14-year-old male patient who exhibited Gorham’s disease in the right mandible, characterized by the loosening of teeth.

## Introduction

Gorham’s disease is a rare and progressive condition that affects the skeletal system, leading to the gradual loss of bone tissue, which is subsequently replaced by vascular and fibrous connective tissue [1,2]. This condition is known by various other names in the medical literature, including phantom bone disease, disappearing bone disease, acute spontaneous bone absorption, hemangiomatosis, lymphangiomatosis, idiopathic osteolysis, and Gorham’s disease [3,4]. The first recorded case was reported by Jackson in 1838, who described a disappearing humerus and coined the term "a boneless arm" [5]. In 1924, Romes documented the disease's occurrence in the jaws [6], while Thoma described complete mandible lysis in 1933 [7]. The disease is often diagnosed following an injury or fracture, with pain being a common initial symptom. In the maxillofacial region, typical symptoms include pain, loose teeth, and pathological fractures [4,8]. Early treatment attempts for maxillofacial vanishing bone disease (VBD) were made by Knolle and Meyer, who used osteogenic substrates such as calcium, phosphate, and vitamin D combined with radiation to manage the condition [9]. In 1974, Booth introduced the use of bone grafting and metal implants to treat pathological fractures caused by VBD [10]. Later, Hirayama pioneered the use of bisphosphonates to slow bone resorption and encourage bone turnover [11].

## Case presentation

A 14-year-old male patient presented with a pathological fracture following the extraction of tooth 46. The pain was intermittent, dull in nature, moderate intensity, and aggravated by eating, but it was relieved on its own. The patient also experienced difficulty eating and speaking. There was no evidence of associated fever, trauma, swelling, sinus opening, or pus discharge [12]. The patient's medical, personal, and family histories were non-contributory [12].

**Figure 1:**
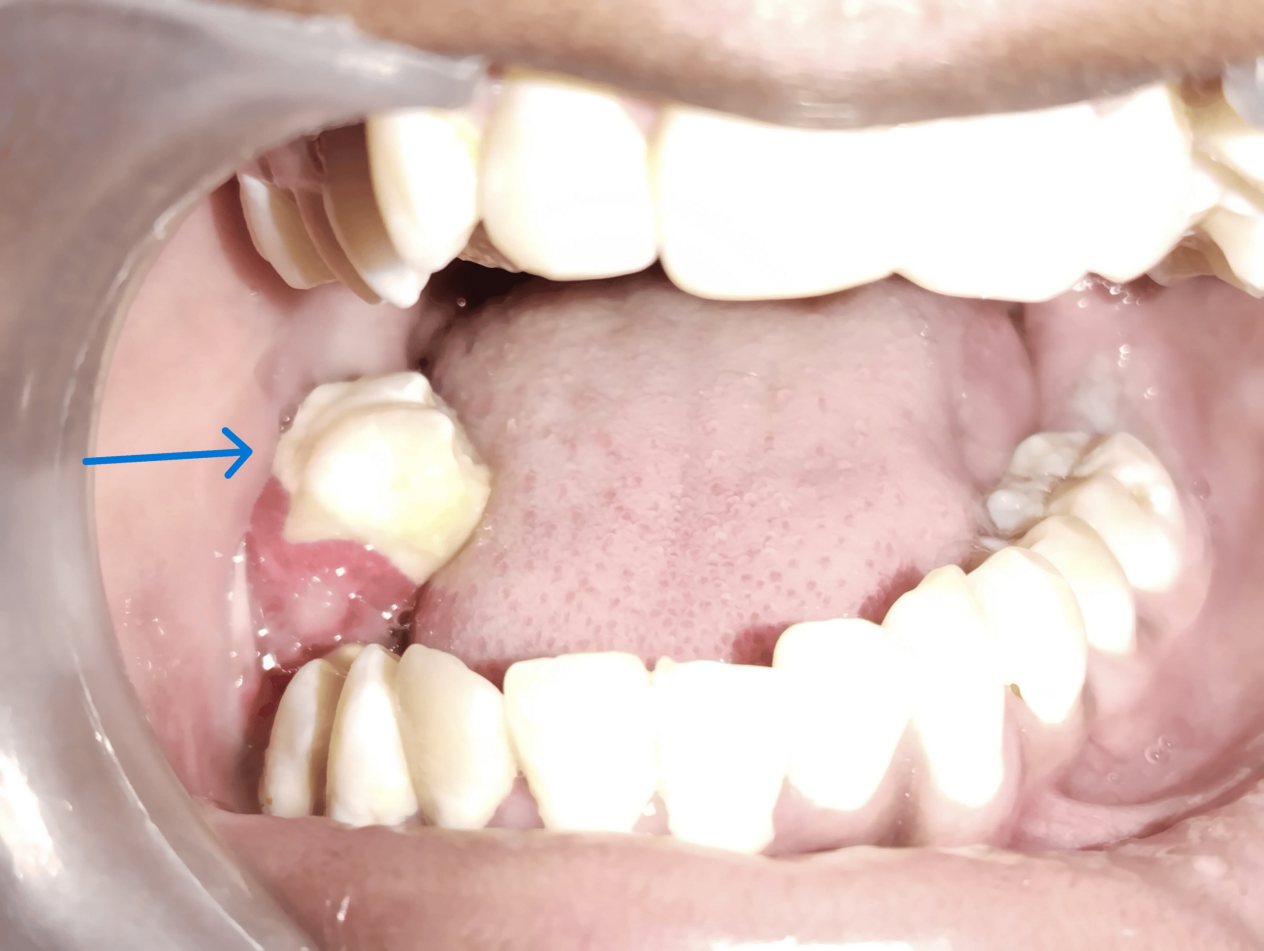
Teeth in the lower right quadrant appeared displaced.

**Figure 2:**
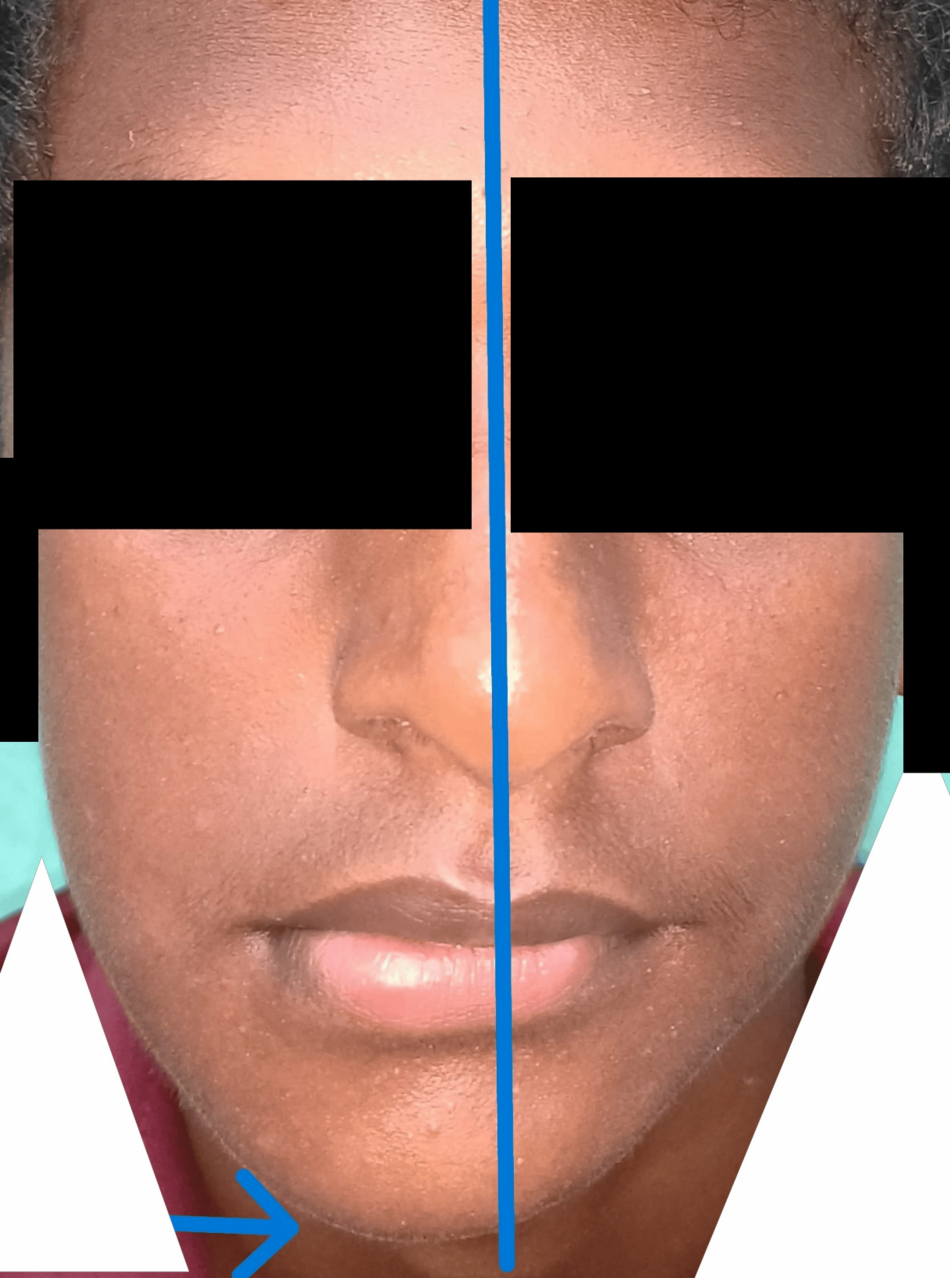
Deviation of the mandible evident toward the ipsilateral side.

**Figure 3:**
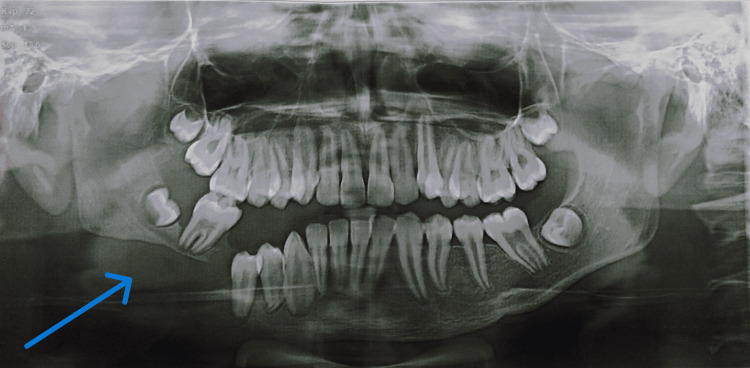
A panoramic radiograph revealed an excessively thin bone rim with a pathological fracture in the right body of the mandible.

**Figure 4:**
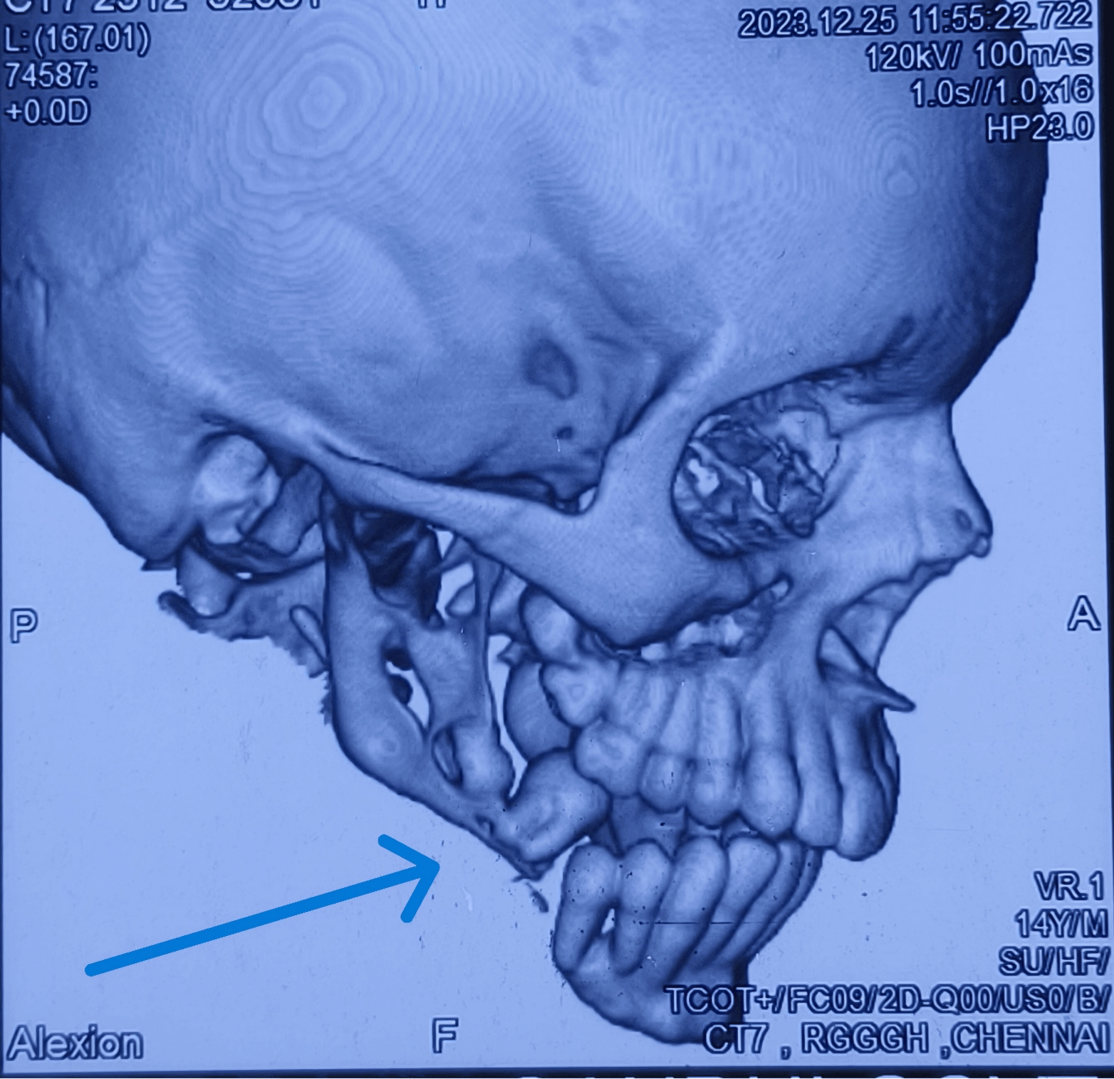
Computed tomography of the head and neck region (3D reconstruction images) showed severe osteolytic changes involving the right mandible.

Intraoral examination revealed that all teeth in the lower right quadrant appeared displaced, and tooth 46 was missing (Figure 1). The maxillary dentition appeared normal for the patient's age. Palpation elicited bleeding on probing, along with grade III mobility of teeth 45 and 47. Reduced thickness of the mandible was noted, with a suspected step deformity in the lower right quadrant posteriorly. Deviation of the mandible was observed towards the ipsilateral side (Figure 2). A panoramic radiograph revealed an excessively thin bone rim with a pathological fracture in the right body of the mandible (Figure 3). Computed tomography of the head and neck region showed severe osteolytic changes involving the right mandible (Figure 4).

Hematological investigations, including acid phosphatase, alkaline phosphatase levels, and serum calcium, were within normal limits, and no endocrine abnormalities were identified [12]. Chest radiography findings were also normal. Based on the patient's history, clinical examination, and radiographic findings, a final diagnosis of VBD of the mandible was made.

Surgical procedure

**Figure 5:**
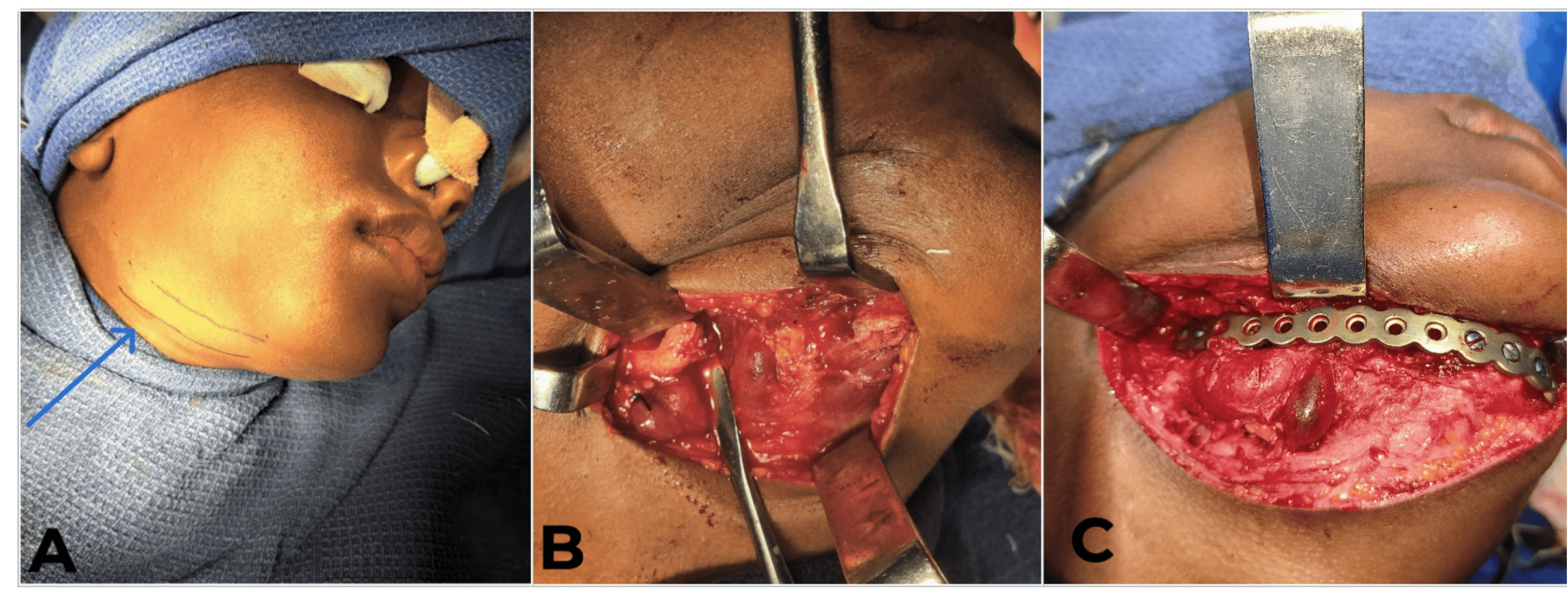
Surgical steps. A: The extraoral submandibular incision marking was done. B: A meticulous dissection was done, and a thorough exploration of the mandible was conducted to assess the extent of the damage. C: The mandible was reconstructed using a load-bearing plate to restore its form and function.

**Figure 6:**
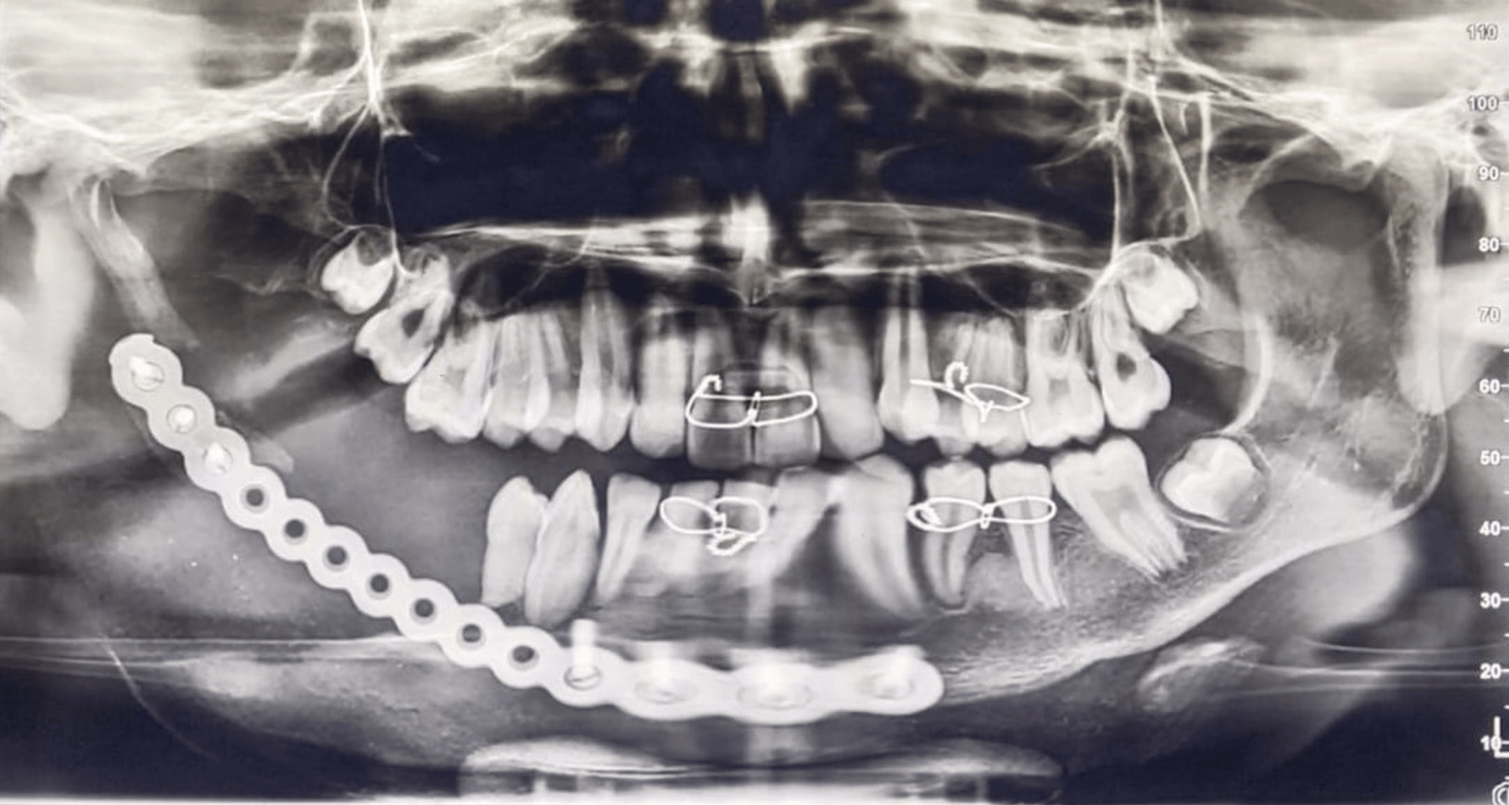
Immediate postoperative OPG. OPG: Orthopantomogram

**Figure 7:**
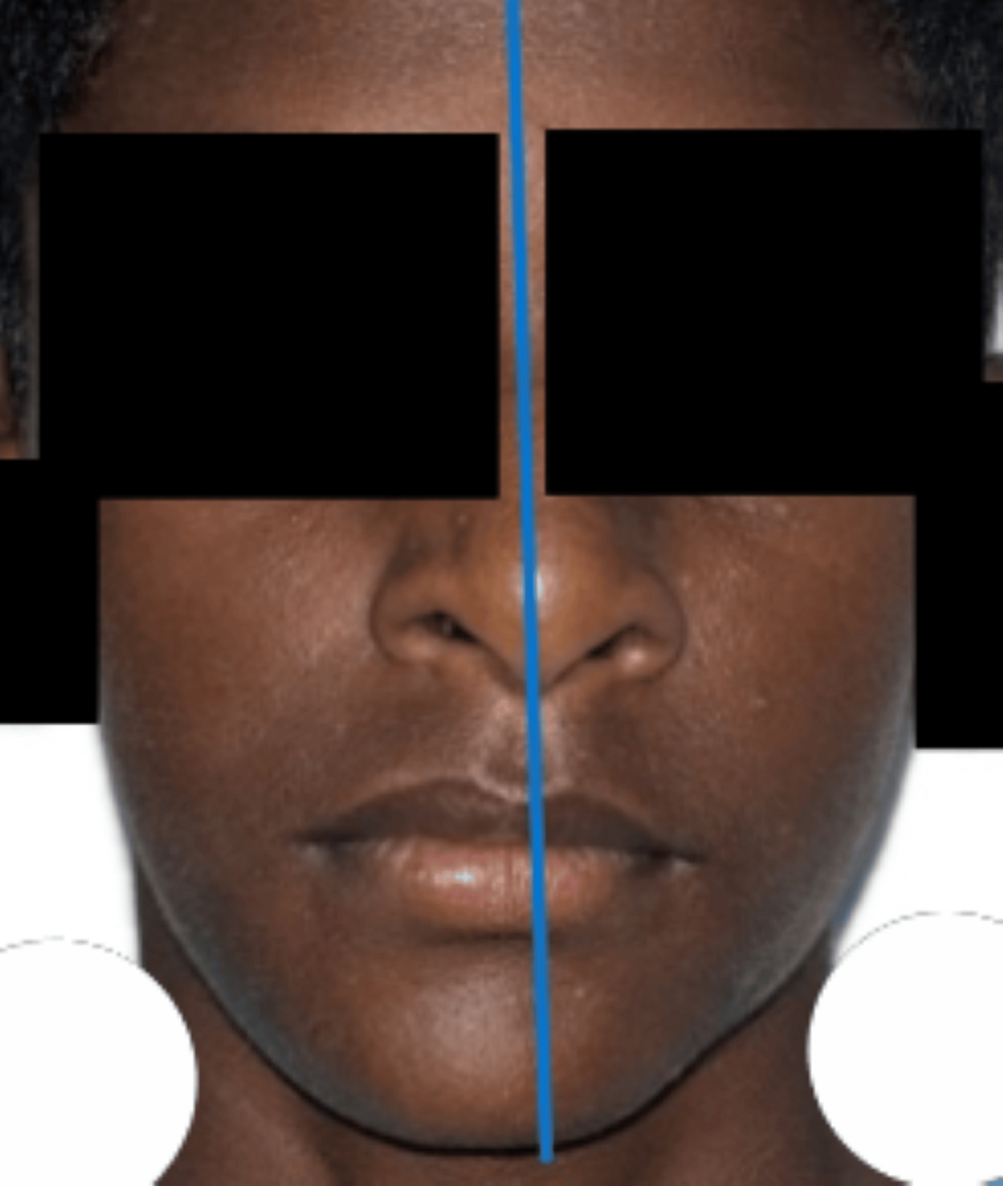
After a four-month follow-up, it was noted that the chin deviation had been reduced.

A right submandibular incision was made to access the pathological fracture located in the right body of the mandible (Figure 5A). The incision was carefully planned to provide optimal exposure while minimizing damage to surrounding structures. Both the facial artery and vein were encountered during dissection and were ligated to prevent excessive bleeding, ensuring a clear operative field. Upon accessing the fracture site, a thorough exploration of the mandible was conducted to assess the extent of the damage (Figure 5B). The pathological fracture in the right body of the mandible was identified, and its alignment was carefully examined. After ensuring proper debridement and removal of any damaged tissue, the mandible was reconstructed using a load-bearing plate (Figure 5C). This plate was secured to restore both the function and strength of the fractured area, ensuring optimal healing and stabilization. After confirming the stability of the mandible and achieving satisfactory hemostasis, the wound was closed in layers. Figure 6 shows the immediate postoperative orthopantomogram. Figure 7 demonstrates that the chin deviation was reduced after a four-month follow-up.

## Discussion

The exact cause of massive osteolysis remains unclear, but ongoing clinical studies aim to enhance our understanding of its underlying causes. Many cases of this condition go unreported, as many general dentists are unfamiliar with the disorder. While massive osteolysis is typically a monocentric disease, it is locally aggressive, with bone loss extending into adjacent soft tissues [12]. Heffez et al. outlined the diagnostic criteria for massive osteolysis, emphasizing several key factors [13]. These include evidence of progressive local bone resorption, with little to no osteoblastic activity and an absence of dystrophic calcification. The lesion is non-expansile and non-ulcerative, with no associated visceral involvement. Radiographically, the condition appears osteolytic in nature. Additionally, the diagnosis requires negative findings for any hereditary, metabolic, neoplastic, immunological, or infectious causes, ensuring that other potential explanations for bone loss are ruled out [12].

The clinical presentation of massive osteolysis varies depending on the area affected. Some patients experience sudden pain, swelling, or pathological fractures, while others report gradual pain, limited movement, and progressive weakness in the affected area [14]. The condition typically does not present with systemic symptoms [15]. In most cases, bone resorption may halt spontaneously, leading to a generally favorable prognosis unless vital structures are involved. Diagnosing massive osteolysis can be challenging, as laboratory tests are often non-specific and do not assist in the diagnosis. Radiographs are typically the most useful tool for identifying the condition. Johnson and McClure first described the radiographic features of massive osteolysis in 1958 [16]. Early stages are characterized by radiolucent areas in the intramedullary or subcortical regions, with well-defined margins. Over time, these areas merge, involving the cortex and leading to gradual bone dissolution, atrophy, fracture, fragmentation, and eventually the disappearance of bone. This process results in the remaining bone tapering or pointing, with surrounding soft tissues atrophying. In the mandible, the resorptive process continues until only small remnants of bone remain.

The differential diagnosis for Gorham's disease includes other causes of osteolysis, such as inflammatory diseases (e.g., osteomyelitis), trauma, endocrine disorders (e.g., hyperparathyroidism), rheumatoid arthritis, skeletal angiomas, angiosarcoma, and other cancers [17]. In our case, the differential diagnosis included Langerhans cell histiocytosis, Papillon-Lefèvre syndrome, and hypophosphatasia, given the extensive gingival lesions and tooth mobility. Management options typically include surgery, radiotherapy, etidronate therapy, and α-2b interferon therapy [18]. Research is also being conducted into the potential use of stem cell therapy for treatment. VBD is a rare disease, with fewer than 400 cases reported in the medical literature to date [19]. In total, 52 cases of maxillofacial VBD have been reported before our report [20].

## Conclusions

Controversy persists regarding the treatment of massive osteolysis due to its rarity and progressive nature. While reconstructive treatments are sometimes employed to restore bone function, their effectiveness remains uncertain. This case report underscores the importance of oral diagnosticians recognizing this disease as a potential cause of bone destruction in the facial skeleton. Massive osteolysis should be considered a rare but important differential diagnosis for facial skeletal lesions.
